# 

**Published:** 1842-06

**Authors:** 


### Recent Publications.

*Précis sur le redressement des dents.* Par J. M. A. Schange, in 8o. plus 8 pl. Paris, 1842.

In the *Bulletin Bibliographique,* of February the 1st, published by Hector Bossange, the above work is announced. Not having yet seen it, we are of course unable to speak of its merits. It is said, however, to be an excellent treatise on the means of preventing and correcting irregularities in the arrangement of the teeth. It also contains some remarks on obturations of the palate. We will notice the work more at large after we shall have received it.

*Meeting in Boston.*—The members of the American Society of Dental Surgeons, have, we presume, all been regularly notified of the time and place of the next meeting, and we hope that the considerations urged by the recording secretary will influence all to make some sacrifices to be present on the occasion.

### COLLABORATORS.

The following gentlemen were elected at the last meeting of the American Society of Dental Surgeons, Collaborators of the American Journal and Library of Dental Science:

| | |
|---|---|
| H. H. HAYDEN, M. D. | Baltimore. |
| ELEAZAR PARMLY, M. D. | New York. |
| EDWARD MAYNARD, M. D. | Washington City. |
| H. M. HAYDEN, M. D. | Baltimore. |
| ELISHA TOWNSEND, | Philadelphia. |
| VERNON CUYLOR, M. D. | Hartford, Ct. |
| ISAAC I. GREENWOOD, M. D. | New York. |
| G. G. BREWSTER, | Portsmouth, N. H. |
| LUDOLPH PARMLY, M. D. | Mobile, Ala. |
| EDWARD TAYLOR, M. D. | Natchez, Miss. |
| C. S. BREWSTER, M. D. | Paris, France. |
| LEONARD KOECKER, M. D. | London, England, |
| LEWIS ROPER, M. D. | Philadelphia. |
| H. S. BURR, M. D. | Philadelphia |
| E. BAKER, M. D. | New York, |
| HORATIO N. FENN, M. D. | Rochester, N. Y. |
| ALEXANDER NELSON, M. D. | Albany, N. Y. |
| B. A. RODRIGUES, M. D. | Charleston, S. C. |
| ——— ROGERS, M. D. | Cincinnati, Ohio. |
| ENOCH NOYES, M. D. | Baltimore. |

---

SUBSCRIPTIONS TO THE SECOND VOLUME.—Our readers will perceive by reference to the Prospectus on the fourth page of the cover, that the terms of subscription are payment in advance. The Journal having become the property of the American Society of Dental Surgeons, the conductors of the publication are required by that body, to see that this condition is strictly complied with; and, they mention it, that those of the subscribers to the first volume who have signified their wish to continue their subscription, without having paid the amount, may not attach any blame to the editors for not furnishing them with it, until they shall have done so.

---

OFFICERS AND MEMBERS OF THE AMERICAN SOCIETY OF DENTAL SURGEONS.—A catalogue of the Officers and Members of this Society has just been published by the Recording Secretary, by order of the same. It is beautifully executed, and in a convenient form for framing. Every member should have one, and they may be procured by application to the above named officer, on parchment, silk, paper or white satin, by transmitting to him one dollar, the price fixed by the Society.

---

DIPLOMAS.—The American Society of Dental Surgeons is now provided with a beautifully executed diploma plate, and will furnish such of its members as desire it, with a parchment diploma. Any member, therefore, who may wish to procure one, will be furnished with the same, by transmitting ten dollars to the Treasurer, or Recording Secretary.

*Receipts of Subscriptions to the Second Volume of the American Journal and Library of Dental Science.*

Ayling, J., Boston,................$5 00
Atkinson, James, Leeds, England,....5 00
Briscoe, James H., Philadelphia,......5 00
Bemis, S. A., Boston,...............5 00
Ball, A., Boston,.....................5 00
Dixon, R. E., Dr.,....................5 00
De Loud, Chas., Wolverhampton, Eng. 5 00
Davis, James A., Lumpkin, Stewart County, Ga.,.................5 00
Geer, J. A., Rev., Liberty, Md.,......5 00
Gidney, Eleazar, Manchester, England, 5 00
Gunning, T. B., New York,..........5 00
Grimme, C., Raleigh, N. C.,.........5 00
Harris, John, M. D., Georgetown, Ky., 5 00
Hayden, Chester, Washington City,...5 00
Helsby, Rich'd, Manchester, England, 5 00
Harris, James M., Philadelphia,.......5 00
Jenks, W. D., Dr., Frederick, Md.,...5 00
Jewett, Wm. H., Bangor, Me.,...... 5 00
Lloyd, Richard, Liverpool, England,..5 00
Middleton, Ellis, Philadelphia,........5 00
Merryman, George, Baltimore,... ...5 00
McCullum, H., Augusta, Ky.,.........5 00
Mackall, L., M. D., Baltimore, Md....5 00
McPherson, James, Glasgow, Scotland, 5 00
Mead, M. B., Providence, R. I.,......5 00
Noyes, Enoch, M. D. Baltimore, ....5 00
Nasmyth, Robert, Edinburg, Scotland, 5 00
Parsons, J. H., Liverpool, England,...5 00
Peak, James M., Cooperstown, N. Y., 5 00
Ross, James C., Dr., Frankfort, Ky....5 00
Smith, J. W., M. D., Amherst, Mass. 5 00

NOTE.—Not having received the names of our subscribers obtained by our London publisher, and many of our agents, we are unable to acknowledge the receipt of their subscriptions to the present volume; we shall do so, as soon as lists shall have been furnished.

### EDITORS' PROSPECTUS
OF THE
### American Journal and Library of Dental Science.

As this Journal has become the organ of the American Society of Dental Surgeons, and as its name has been somewhat modified, it may be considered as having entered upon a new period of its existence; and its former publishers congratulate themselves and the profession on its good fortune. Its perpetuity is now considered as settled. The profession will always need a periodical devoted to its interests, and who is better able to conduct it, than the only regularly organized and extensive association of dental surgeons in the world. We repeat, that we congratulate ourselves and our professional brethren on an event so auspicious to the science of dental surgery, as the formation of a national society and the transfer of the journal to that body. The work can now be regularly issued, whether its circulation be co-extensive with the profession or not. If the subscription should be limited, the issues can be made to correspond with the demand, so that the expense to the society may be kept wholly within its means.

The American Journal and Library of Dental Science, is now proposed to take an independent ground, and will hereafter solicit no favours from those members of the profession who are opposed to the dissemination of periodical information. To others who are favourably inclined to a community of knowledge, this journal will not fail to commend itself, as one of the most efficacious means of exposing the impositions of quackery, and imparting to honorable pretensions that kind of information which their usefulness and talents should secure.

The journal will be regularly issued on the first of December, March, June and September, annually, at the enhanced price of five dollars per volume, payable in all cases in advance. The price of subscription may be paid to the treasurer of the society, Dr. E. Baker, No. 6 Warren-st. New York, or to either of the editors, or any of the publishers, collaborators or acknowledged agents o the journal. Advance payment is required by a special resolution, passed at the last meeting of th society, and is necessary to protect it from pecuniary loss, a neglect of which has involved th publishers of the first volume in unexpected embarrassment, if not in ultimate loss.

To save postage, the agents in all the large cities of the United States, and Great Britain, will b furnished with copies to supply subscribers, by such conveyance as they shall prescribe. An where it can be done, even individuals may receive their numbers by private conveyance if they choose.

With these few explanatory remarks, we commit our work to its coming destiny, after assuring the profession that no efforts will be spared to render it every way worthy of the great subject to which its pages will be exclusively devoted.

CHAPIN A. HARRIS,
SOLYMAN BROWN.

---

Besides the Collaborators, the following gentlemen are regularly authorized

### AGENTS OF THIS JOURNAL.

Bradbury & Soden,........Boston.

F. B. Chewning,............Richmond, Va.

J. P. Laird, M. D............Columbus, Ga.

Geo. Washington Parmly, New Orleans.

G. McDonald, M. D .......Macon, Ga.

Blandin & Avery,.........Columbia, S. C.

Eleazar Gidney,...........Manchester, Eng.

J. Allen,...................Cincinnati, Ohio.

A. B. Hayden, D.D. S.....Savannah, Geo.

Drs. Goddard and Ferguson, Louisville, Ky.

John Harris, M. D..........Georgetown, Ky.

Nicholas Clute,...........Louisville, Ky.

J. N. Frink,................Portland, Me.

E. J. Dunning,...............Buffalo, N. Y.

Benj. Strickland,...........Cleveland, Ohio.

B. B. Brown, M. D..........St. Louis, Mo.

W. R. Scott, D. D. S.........Raleigh, N. C.

S. M. Shepherd,............Petersburg, Va.

W. W. H. Thackston, D. D. S. Farmville, Va.

